# Spatial Analysis of Sleeping Sickness, Southeastern Uganda, 1970–2003

**DOI:** 10.3201/eid1205.051284

**Published:** 2006-05

**Authors:** Lea Berrang-Ford, Olaf Berke, Lubowa Abdelrahman, David Waltner-Toews, John McDermott

**Affiliations:** * University of Guelph, Guelph, Ontario, Canada;; † University of Veterinary Medicine, Hannover, Germany;; ‡ Makerere University, Kampala, Uganda;; § International Livestock Research Institute, Nairobi, Kenya

**Keywords:** Trypanosomiasis, African sleeping sickness, Uganda, Africa, spatial distribution, historical review

## Abstract

Sleeping sickness reemerged in southeastern Uganda in the 1970s and remains a public health problem. It has continued to spread north into new districts, and gaps remain in the understanding of the causes of its spread and distribution. We report the distribution and magnitude of sleeping sickness in southeastern Uganda from 1970 to 2003. Data were collected from records of the Ugandan Ministry of Health, individual sleeping sickness treatment centers, and interviews with public health officials. Data were used to develop incidence maps over time, conduct space-time cluster detection analyses, and develop a velocity vector map to visualize spread of sleeping sickness over time in southeastern Uganda. Results show rapid propagation of sleeping sickness from its epicenter in southern Iganga District and its spread north into new districts and foci.

## Abstract 2

Disease will likely spread into central Uganda.

Sleeping sickness is the human form of African trypanosomiasis (caused by *Trypanosoma* spp.), a protozoal parasitic disease affecting humans, livestock, and many sylvatic species in sub-Saharan Africa. It is transmitted by the tsetse fly vector (*Glossina* spp.) and in cattle is a serious constraint to livestock development in sub-Saharan Africa (*1**–**3*).

The acute form of sleeping sickness, which is caused by *Trypanosoma brucei rhodesiense* and is predominant in eastern and southern Africa (*4**–**6*), is present in southeastern Uganda (Figure 1). Sleeping sickness is a serious public health problem in this region; epidemics have occurred in 1901–1915, 1940–1946, and 1976–1989 (*3*). More recently, spread of sleeping sickness into areas previously thought to be free from the disease has highlighted gaps in the ability of current research to explain and predict the distribution of infection (*7*).

**Figure 1:**
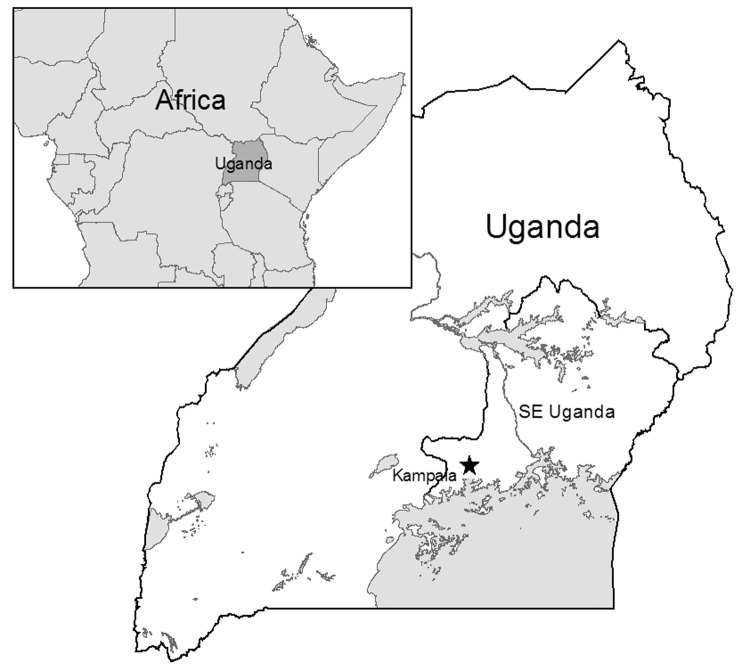
Location of the study site in southeastern (SE) Uganda. The star indicates the capital of Kampala. Inset shows surrounding countries in Africa.

In 1976, an outbreak was detected in Luuka County in western Iganga District, outside the traditional fly zone. This was the beginning of an extensive epidemic that eventually spread throughout southeastern Uganda. This outbreak occurred during a time of great political instability and civil conflict in Uganda, which contributed to a reduction of resources and services for sleeping sickness (*8*). Although the incidence of sleeping sickness decreased in southeastern Uganda in the early 1990s, it continues to persist and spread in 2005. An outbreak was detected for the first time in Soroti District in 1998 (*7*), followed by continued spread north into Kumi, Kaberamaido, and Lira Districts (*9**,**10*). Historical analyses of sleeping sickness in southeastern Uganda can improve disease control by increasing understanding of the context and trends of the disease, as well as identifying variables associated with these trends. Additionally, historical analyses may validate hypothesized processes.

We describe and characterize the spatial distribution of *T*. *b*. *rhodesiense* sleeping sickness in southeastern Uganda for a 34-year epidemic period (1970–2003). We hypothesize that sleeping sickness in southeastern Uganda is driven by 2 dominant processes. In process A, in regions where disease occurs or has recently occurred, localized outbreaks are triggered by processes that increase tsetse populations or by changes that increase human-tsetse contact. In process B, in regions where disease has not recently occurred, spread is facilitated by movement of infected livestock into uninfected regions.

## Methods

### Study Area

The study area in southeast Uganda in eastern Africa (Figure 1) is subdivided into 17 districts, 46 counties, and 254 subcounties. The region has an area of ≈55,000 km^2^ and a population of ≈9 million (*11*). Thirteen percent live in the capital of Kampala (*11*), and the remainder live in predominantly rural areas dominated by livestock and subsistence farming (*12**,**13*).

### Data Collection

Cross-sectional sleeping sickness data from 1970 to 2003 were collected retrospectively in 2004 to identify case counts and measures of disease magnitude per subcounty per year. Data were collected for all available records of sleeping sickness patients in southeastern Uganda. Data availability and reliability varied between years on the basis of quality of surveillance and primary data collection, as well as availability of records and recall bias for secondary data collection. Reliability of data for 1986 to 2003 was considered moderate to high, but reliability of data for 1970 to 1986 was low to moderate. Evaluation and review of data by public health officials concluded that information on disease prevalence and absence was reliable for most years, but measures of disease magnitude were less reliable before 1986.

**Figure 2:**
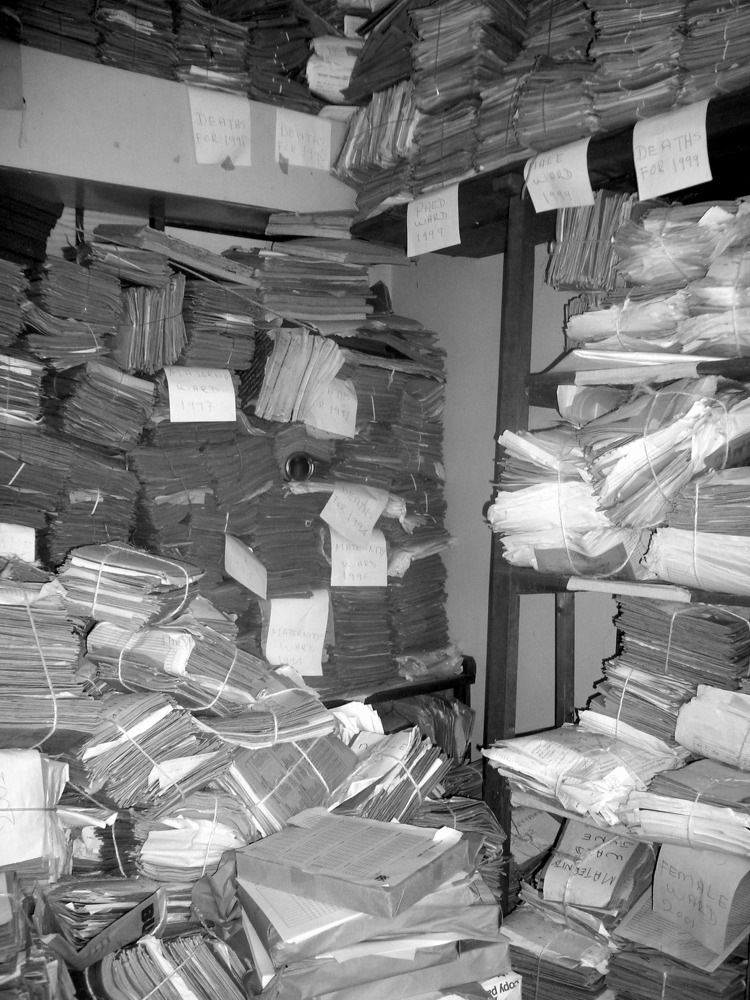
Records room at Bugiri Hospital sleeping sickness treatment center, Uganda.

Since 1999, sleeping sickness data summaries have been provided by the National Sleeping Sickness Control Program at the Ugandan Ministry of Health. For data before 1988, no centralized collection of records exists beyond district summaries; sleeping sickness case data remain in records at individual treatment centers. Data before 1988 were collected retrospectively in 2004 during visits to all treatment centers active in the 1980s. In many cases, record books were poorly stored, damaged, had lost pages, or were missing (Figure 2). Case definition was based on the primary diagnosis; all cases recorded in record books were included in this study. Cases were assigned to a year based on the patient's date of admission.

Gaps in the dataset increased before 1986. Limited data were available for the late 1970s, and no quantitative data were available for the early- to mid-1970s. Interviews were conducted with public health officials to complement and extend 1970s and 1980s data. These officials were chosen by identifying Ugandans actively involved in senior positions in sleeping sickness prevention and control from 1970 to the present and those who could be contacted. They included veterinary and public health managers at the National Ministry of Health or District Medical or Veterinary Office levels. Interviews were used to verify data for the 1980s and to classify disease magnitude for the 1970s.

Sleeping sickness magnitude was classified into 1 of 5 categories for each subcounty for each year: 1) no cases, 2) preepidemic (1–4 cases per year per subcounty), 3) low epidemic (5–15 cases per year per subcounty), 4) high epidemic (16–100 cases per year per subcounty), and 5) extreme epidemic (>100 cases per year per subcounty). These thresholds were based on anecdotal guidance from preliminary interviews and defined to facilitate standardized definitions of magnitude during subsequent interviews. Interviewer information was compared to available sleeping sickness records and the literature. These results were used to develop a classification database of disease magnitude by subcounty and year. Resulting data and maps were presented to informants for discussion and validation at follow-up interviews in 2005.

The boundaries of political regions changed greatly during the 34-year study period. Aggregation of subcounty data reduced the number of subcounties from 254 in 2004 to 225 for the current study period. Temporal resolution of data is consistent, and aggregation of 29 subcounties is assumed to have little effect on overall analyses.

### Data Analysis

Case counts from 225 subcounties in southeastern Uganda for 34 years (1970–2003) were aggregated into 5 temporal periods for descriptive and geographic cluster analysis on the basis of epidemic progression and data availability: 1970–1975, preepidemic; 1976–1979, epidemic increase; 1980–1888, epidemic peak; 1989–1997, epidemic decrease; and 1998–2003, epidemic tail. Mean case counts per subcounty per year were calculated for periods in which case counts were available (1980–1988, 1989–1997, and 1998–2003). For earlier periods (1970–1975 and 1976–1979), only ordinal data were available. Therefore, data midpoints were calculated by using the mean of the maximum and minimum ordinal values rounded to the nearest whole number in the direction of the mode. These data were used to develop maps averaging the annual incidences of sleeping sickness for each subcounty during the interval period. Averages of annual incidence for intervals after 1980 were reclassified as sporadic (<5 cases/year), low epidemic (5–15 cases/year), or high epidemic (>15 cases/year) to match ordinal data categories for 1970s data.

To identify clusters of sleeping sickness in southeastern Uganda from 1970 to 2003, the space-time scan statistic (*14*) was used (SaTScan version 5.1 software for spatial and space-time scan statistics available from http://www.satscan.org/ [Kulldorff, Boston, MA, USA and Information Management Services Inc., Silver Springs, MD, USA]). The incidence proportions of the 225 subcounties were assumed to follow a Poisson distribution according to the underlying population size. Cluster analysis results include space-time clusters with no geographic overlap of clusters allowed and a maximum allowable cluster size of 50% of the population. Space-only and time-only clusters were excluded. Primary and secondary clusters at a significance level of α = 5% are reported.

Sleeping sickness data were used in the form of case counts per year per subcounty for post-1980 records. For 1970s data, recorded values represent ordinal data (i.e., low, medium, high) rather than case counts. These were transformed to case counts by applying the ordinal minimum value to each record. Population data are based on the 1980, 1991, and 2002 population censuses for Uganda (*11*). The first 3 analysis periods (1970–1988) used 1980 census records for population counts. Analyses for the periods 1989–1997 and 1998–2003 are based on 1991 and 2002 census records, respectively.

A vector velocity map (*15*) of sleeping sickness spread was developed by using trend surface analysis (TSA) (*16**,**17*). TSA is a global smoothing method using polynomials in geographic coordinates, as defined by the central point of each subcounty polygon. In this case, a trend surface of the year of the first reported sleeping sickness case for each subcounty was used to explore and identify diffusion patterns and corridors of spread over time.

The year of the first recorded case was identified for subcounties in the database. Eighty-nine of 225 subcounties with no recorded cases in the 1970–2003 study period were excluded. The x- and y-coordinates of subcounty centroids were calculated from a UTM projection shapefile of southeastern Uganda using ArcMAP (ArcGIS 9, Environmental Systems Research Institute, Redlands, CA, USA). Least square regression using linear, quadratic, cubic, and higher-order polynomials of the x- and y-coordinates to predict year of first reported case was conducted in R (Foundation for Statistical Computing, Vienna, Austria, available from http://www.R-project.org). Partial differential equations (Δyear/ΔX and Δyear/ΔY) were derived from the fitted model, giving a vector of the magnitude (slope) and direction for each location. The square root of the slope equates to the velocity of diffusion.

## Results

### Epidemic Curve

**Figure 3:**
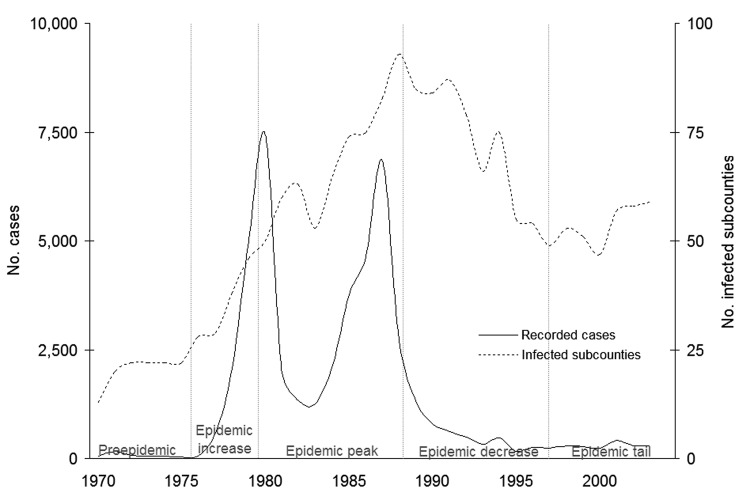
Number of sleeping sickness cases and infected subcounties, southeastern Uganda, 1970–2003. Number of recorded cases refer to totals for southeastern Uganda. Sources: 1970–1971, D.B. Mbulamberi, unpub. data; 1972–1975 (*18*); and 1976–2001 (Ministry of Health, 2004).

Figure 3 shows the epidemic curve for 1970 to 2003 in southeastern Uganda, as well as a curve of the total number of subcounties infected per year. The latter curve gives an indication of the spatial extent of the disease in the region, while the former indicates the magnitude of the epidemic. The dramatic decrease in incidence in 1982 and 1983 is related to both German Red Cross intervention in 1980 in the Luuka County region (*19*) (D.B. Mbulamberi, pers. comm.) and reduced surveillance in 1982 and 1983. The number of cases and infected subcounties decreased in the 1990s. In contrast to the decrease in incidence, however, the number of infected subcounties remains well above preepidemic levels.

### Incidence Maps and Cluster Detection

**Figure 4:**
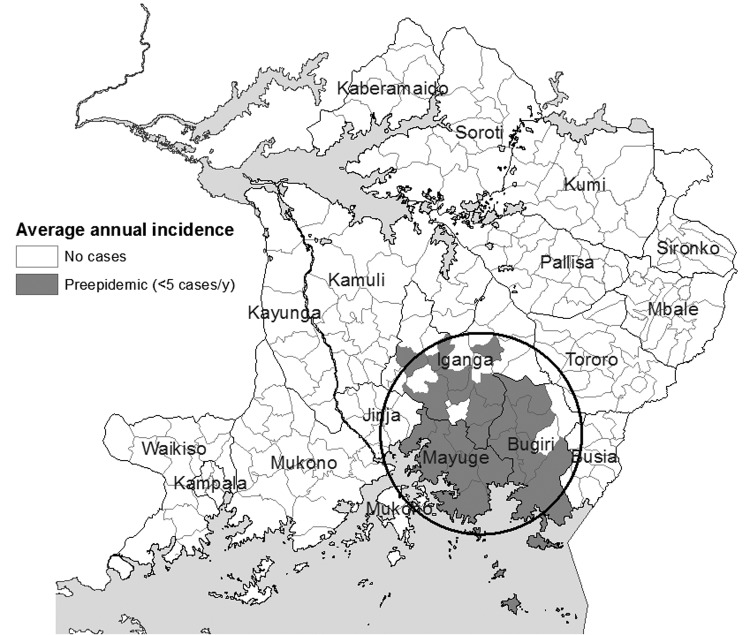
Sleeping sickness incidence, southeastern Uganda, 1970–1975, by subcounty. Circle indicates a significant space-time cluster at the 95% confidence level, as detected by the space-time scan test. See Table for scan test results.

**Figure 5:**
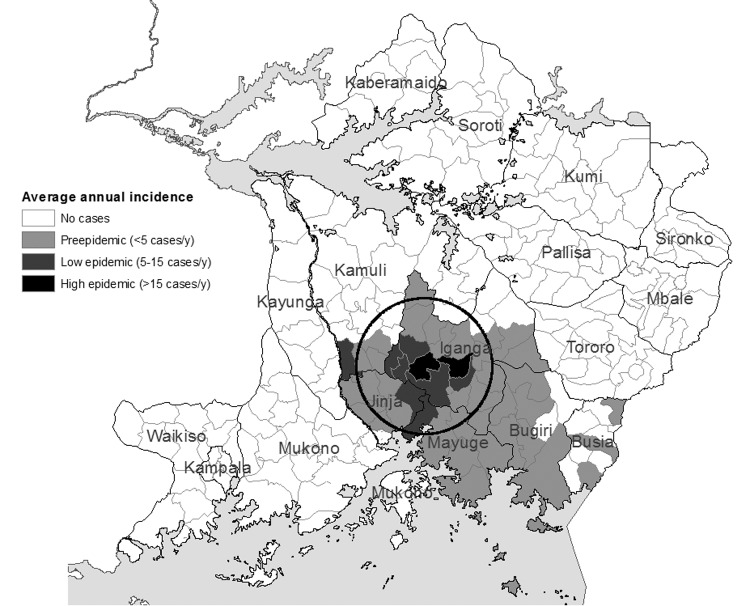
Sleeping sickness incidence, southeastern Uganda, 1976–1979, by subcounty. Circle indicates a significant space-time cluster at the 95% confidence level, as detected by the space-time scan test. See Table for scan test results.

**Figure 6:**
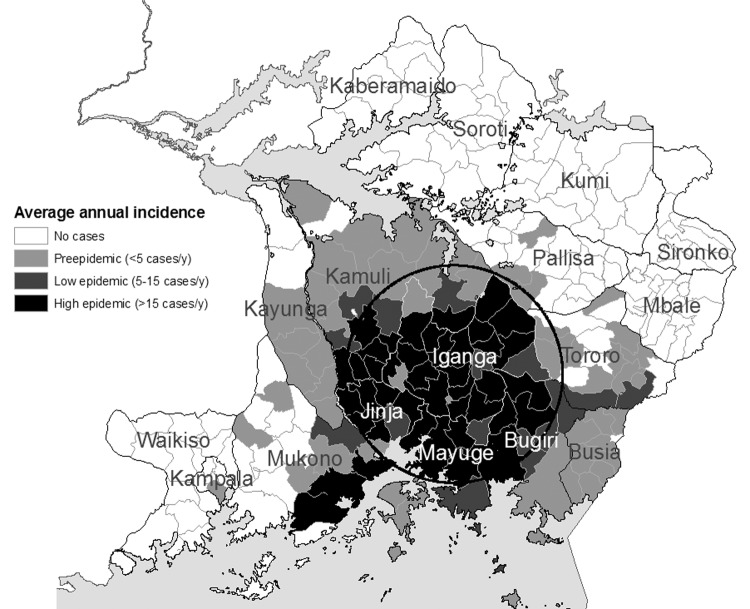
Sleeping sickness incidence, southeastern Uganda, 1980–1988, by subcounty. Circle indicates a significant space-time cluster at the 95% confidence level, as detected by the space-time scan test. See Table for scan test results.

**Figure 7:**
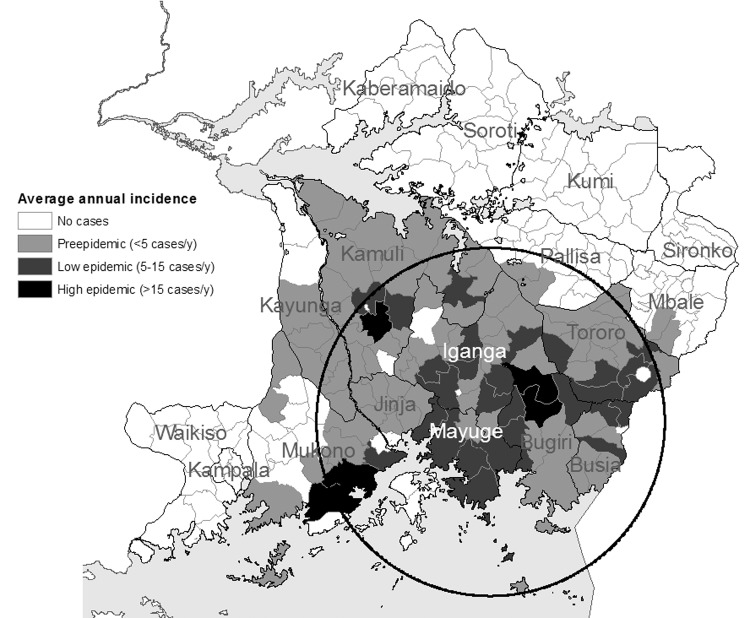
Sleeping sickness incidence in southeastern Uganda, 1989–1997, by subcounty. Circle indicates a significant space-time cluster at the 95% confidence level, as detected by the space-time scan test. See Table for scan test results.

**Figure 8:**
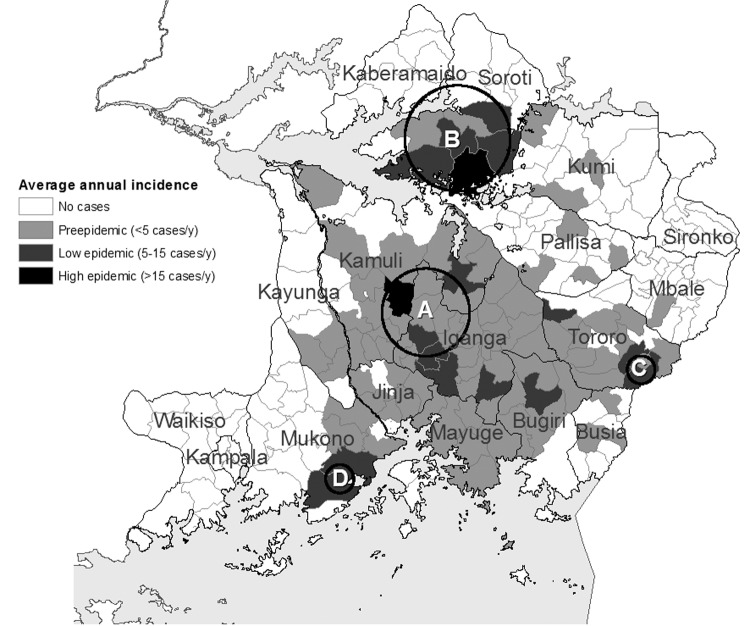
Sleeping sickness incidence in southeastern Uganda, 1998–2003, by subcounty. Circles indicate significant primary (A) and secondary (B, C, and D) space-time clusters at the 95% confidence level, as detected by the space-time scan test. Letters correspond to cluster results in Table. See Table for scan test results.

Figures 4,5, 6,7, and 8 show maps of the average annual sleeping sickness incidence (*T*. *b*. *rhodesiense*) per subcounty in each of the 5 study periods. Legends for the 5 maps are consistent. Each map includes the location of significant (α = 5%) primary and secondary space-time clusters. Results of cluster detection analyses are discussed below for each interval period and are summarized in the Table. Dominant trends in cluster results were insensitive to maximum cluster size.

**Table:** Cluster detection of sleeping sickness, southeastern, Uganda, 1970–2003

Interval (cluster)	Districts in most likely cluster	Cluster date	No. observed cases	No. expected cases	Relative risk*	p value	Cluster radius (km)
1970–1975	Mayuge, Bugiri, and southern Iganga	1973–1975	63	8	8.3	0.0001	41
1976–1979	Northwest shift to include northern Mayuge, Iganga, Jinja, and southeastern Kamuli	1978–1979	311	23	13.5	0.0001	29
1980–1988	Wider extent, including Mayuge, Bugiri, Iganga, Jinja, and southern Kamuli	1985–1988	13,943	1,865	7.5	0.0001	45
1989–1997	As above, plus Tororo, Busia, eastern Mukono, southern Kayunga, and southern Pallisa	1989–1992	3,176	869	3.7	0.0001	74
1998–2003 (A)†	Northwestern Iganga (Luuka county) and southern Kamuli	1999–2001	331	26	12.6	0.0001	19
(B)†	Soroti	2001–2003	263	21	12.5	0.0001	22
(C)†	Tororo (Osukulu subcounty)	2001–2002	89	7	12.9	0.0001	6
(D)†	Mukono (subcounties of Buikwe, Buyikwe, Najja, Ngogwe, and Ssi)	1998	50	4	12.5	0.0001	0‡­_­_

*Observed no. cases/expected no. cases. †Multiple clusters were identified during 1998–2003. Letters correspond to cluster labels in Figure 8. ­­_­_‡Cluster included only 1 observation representing 5 merged subcounties.

### 1970–1975: Preepidemic

Figure 4 shows the distribution of sleeping sickness that approximates the preepidemic zone of traditional and sporadic infection during the 1960s. Most subcounties are reported as having only a few cases per year. A significant space-time cluster was identified in the area that included the districts of Mayuge, Bugiri, and southern Iganga for 1973–1975 (Figure 4, Table). These cluster results reflect the beginning of incidence increase in these subcounties in the latter half of this period.

### 1976–1979: Epidemic Increase

Figure 5 shows both an increase in incidence of sleeping sickness along the Iganga/Mayuge/Jinja District borders as well as outward spread of the disease. These processes characterize the onset and increase of the sleeping sickness epidemic in 1976. A 1978–1979 space-time cluster (Figure 5, Table) of smaller size is identified northwest of the cluster for the previous period. The cluster is identified for the later years of the interval, indicating early epidemic onset and propagation, while the smaller radius of the 1978–1979 cluster reflects increased incidence at the epicenter along the Iganga border with Mayuge and Jinja.

### 1980–1988: Epidemic Peak

Figure 6 shows an extensive increase in both incidence and distribution of sleeping sickness that characterized this peak period of the epidemic. Detection analysis identified a cluster in 1985–1988 (Figure 6, Table) located in the same vicinity as those seen in the 2 previous intervals. The 1985–1988 cluster, in addition to the regions in the 1970s clusters, encompasses areas of Jinja, northern Iganga, and southern Kamuli Districts, indicating continued spatial spread.

### 1989–1997: Epidemic Decrease

Figure 7 shows the average annual incidence of sleeping sickness for the period 1989–1997. A decrease in overall incidence can be observed in conjunction with continued spatial spread. Cluster detection identified a cluster for 1989–1992 that encompassed the same areas as previous clusters, as well as the Districts of Tororo, Busia, and eastern Mukono (Figure 7, Table). In contrast to the previous periods, a space-time cluster was identified in the first years of the period. This finding reflects a shift in the epidemic from progression to regression. The larger spatial size of the cluster, however, indicates continued spread into new areas (Figure 7). Areas of increased incidence are generally shifted east.

### 1998–2003: Epidemic Tail

Figure 8 shows the distribution of sleeping sickness incidence for the period 1998–2003. The overall incidence of disease decreased in the southern districts, and the epidemic was characterized by pockets of disease. In addition, the disease was observed for the first time in Soroti District in the north of the study area (cluster B, Figure 8). Cluster detection was consistent with this distribution of outbreak pockets and foci. Four small clusters were detected (Figure 8, Table). Cluster A was detected for 1999–2001 in the subcounties along the border of Iganga and Kamuli Districts. Cluster B identified a new outbreak focus in Soroti District in 2001–2003, where cases were first recorded in 1998. This cluster reflects the increase in incidence in Soroti to the end of the study period. Although incidence in Tororo District peaked around 1990, small outbreak resurgence in Bugongi and Osukuru subcounties in 2001 and 2002 resulted in cluster detection in Tororo District (cluster C, Figure 8, Table) (*20*). A fourth, smaller, cluster was detected in the subcounties of Buikwe, Buyikwe, Najja, Ngogwe, and Ssi in 1998 (cluster D, Figure 8), which experienced a resurgence of incidence since an earlier peak in 1991.

### Trend Surface Analysis

**Figure 9:**
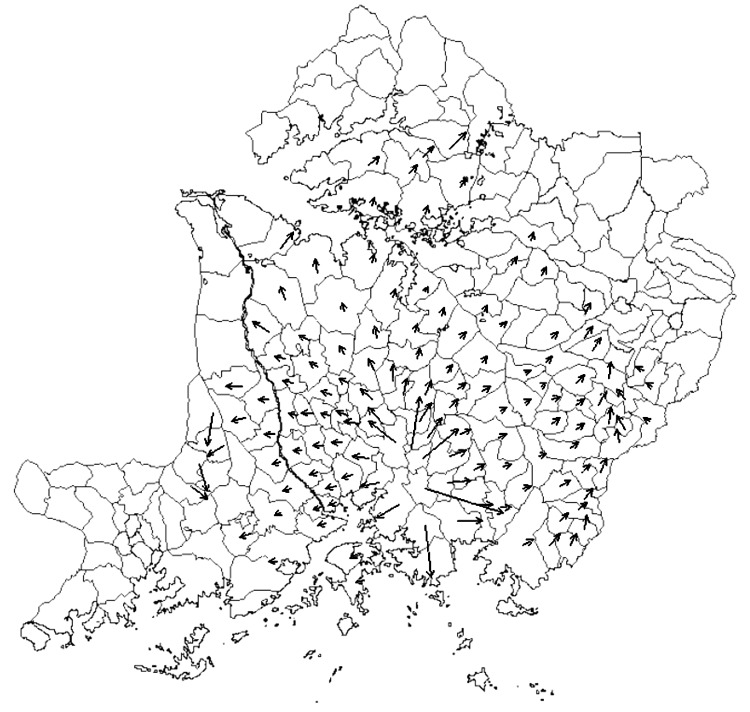
Velocity vectors (arrows) for the spread of sleeping sickness between subcounties in Uganda. Arrow length is proportional to velocity of spread.

The results from trend surface analysis are summarized in a velocity vector map (Figure 9). The velocity and direction of diffusion for each coordinate location were mapped to show the movement and instantaneous rate of *T*. *b*. *rhodesiense* sleeping sickness diffusion in southeastern Uganda over the study period. TSA with high-order polynomials is sensitive to data anomalies at the edge of the study area (*15*). Less data are available at the study area boundaries; velocity vector size and direction are therefore less reliable and may not be accurate at the edge of the study area. For these reasons, 9 velocity vectors were removed from the vector diffusion map (Figure 9).

The average velocity of sleeping sickness spread over 34 years in southeastern Uganda from 1970 to 2003 was 5 km/year. Velocity of movement was highest early in the epidemic (Figure 9), when sleeping sickness spread out of its primary focus in southern Iganga District. The epidemic diffused outward in a relatively constant sphere of diffusion from this epicenter. A corridor of movement can be observed on the eastern fringe of the study area, moving through Busia and Tororo Districts. However, this fringe area should be interpreted with caution because of potential edge effects. The disease moved distinctly north and east into Soroti District. Areas of most rapid spread appear to be the extensions of these 2 corridors north from Soroti and Tororo. These results are consistent with recent detection of cases in the districts of Kumi, Kaberamaido, and Lira (*9**,**10*) adjacent to or north of Soroti. Velocity vectors also showed spread west and east. However, Figures 4–8 suggest that much of this horizontal diffusion occurred before the 1990s.

## Discussion

The reliability of data for the 1970s and 1980s is subject to detection and recording bias associated with periods of passive surveillance, missing record books, and recall bias of interviewees. The creation of additional treatment centers over the study period and differential quality of diagnostic and treatment facilities throughout the study area may contribute to spatial bias in the data. Odiit et al. (*20**–**22*) discuss the potential for misdiagnosis of cases, selective entry bias around treatment centers, and underdetection of sleeping sickness. Aggregation of cases by subcounty reduces the potential for clustering around individual treatment centers, and unless differential misdiagnosis occurs, it will not critically affect the spatial patterns seen. The data must be interpreted with caution in the context of data reliability and potential biases. Results should be considered exploratory and descriptive; data are not appropriate for direct causal inferences. The results are, however, useful for characterizing broad trends; where historical trends in processes observed are consistent with hypotheses, results can inform current and future research.

*T*. *b*. *rhodesiense* sleeping sickness in southeastern Uganda from 1970 to 2003 followed a pattern of radial spread from its center in southern Iganga District. From 1976 to the 1990s (Figures 4, 5, 6 and 7), the epidemic trend coincided with civil unrest and political instability in the country. The increase in the epidemic (1976–1979) occurred at a time of increasing political and economic instability, while the peak epidemic period (1980–1988) occurred during the height of political and economic collapse. The decrease in the epidemic (1989–1997) also coincides with increasing stabilization of politics and civil unrest in Uganda. The epidemic trend observed is consistent with our hypothesis (process A) that incidence increases in regions with a history of infection because of changes in human-vector exposure that push the probability of transmission above the required threshold for focal outbreaks. Uganda in the 1970s and 1980s experienced extensive internal displacement of the rural population, illegal human and cattle movements, growth of favorable tsetse habitats on cotton and coffee plantations, and collapse of sleeping sickness prevention and control activities (*8**,**19**,**23*). These events likely contributed to increased human-vector contact and sleeping sickness transmission in the districts around the preepidemic zone of infection.

After the decrease in the epidemic in the 1990s, new outbreaks have been observed in Soroti (1998, Figure 8), Kaberamaido, Kumi, and Lira (2004–2005) Districts (*7**,**9**,**10*). The introduction of the parasite into Soroti District has been linked to cattle restocking from infected southern districts (*7*). Whether more recent spread into new districts is related to cattle movements is unclear. Postepidemic spread into previously uninfected and peripheral districts since the late 1990s is consistent with our hypothesized second process, which is characterized by parasite spread into new areas through movements of livestock vector. Continuing civil conflict near and within these areas is of particular concern. Once established in new regions, processes of transmission may change from introduction of parasites through cattle movements (process B) into proliferation and continued transmission through increased vector-human exposure resulting from effects of civil conflict (process A). The observed historical trends in sleeping sickness, in the context of our hypotheses, support the likelihood of continued spread of *T*. *b*. *rhodesiense* north from newly infected regions in central Uganda.

Figure 3 suggests that while the number of recorded cases remains low, those cases are coming from an increasing large area. Decreased sleeping sickness surveillance systems in Uganda (D.B. Mbulamberi, pers. comm.) may be missing undetected increases in cases while still detecting infection at the subcounty level. The likelihood of such detection bias is unclear, although a similar difference between recorded cases and recorded subcounties infected preceded the epidemic increase in 1976 (Figure 3). Sleeping sickness is a highly focal disease often characterized by distinct outbreaks in a specific area or village. This outbreak pattern has been smoothed by aggregation of cases to the subcounty level. In spite of the highly focal nature of sleeping sickness, the results suggest a pattern of observable, continuous, and potentially predictable spread of *T*. *b*. *rhodesiense* sleeping sickness in Uganda when data are smoothed to the subcounty level.

The description and characterization of historical reemergence of sleeping sickness in southeastern Uganda can be used to guide and complement research into the causal processes determining the observed patterns of incidence and spread. These patterns are consistent with our hypotheses of 2 dominant processes of sleeping sickness transmission in southeastern Uganda. First, in regions where disease currently occurs or has recently occurred, localized outbreaks are triggered by changes in vector-human exposure or vector numbers, which push the probability of transmission above threshold levels. This process was observed around the traditional infection zone in southeastern Uganda during the 1976–1990 epidemic. Second, in regions where disease has not recently occurred, spread is facilitated by transmission of the parasite thorough livestock. This is currently being observed in the spread of infection to districts in central Uganda that were not infected during the previous epidemic.

Conclusions support further research and intervention related to parasite transmission through cattle movements and potential changes in vector-human exposure in central Ugandan districts. Such analyses are particularly relevant in the context of continued spread of *T*. *b*. *rhodesiense* sleeping sickness in Uganda, potential merging with *T*. *b*. *gambiense* subspecies in northwest regions (*24*), and ongoing civil unrest in north-central regions.
